# Pennsylvania College of Dental Surgery

**Published:** 1859-05

**Authors:** 


PENNSYLVANIA COLLEGE OF DENTAL SURGERY.—The graduating class of this young and flourishing institution was larger during the past session than that of any previous year. The number was 25, while that of the matriculates was 48.

